# Design, structure prediction and molecular dynamics simulation of a fusion construct containing malaria pre-erythrocytic vaccine candidate, *Pf*CelTOS, and human interleukin 2 as adjuvant

**DOI:** 10.1186/s12859-016-0918-8

**Published:** 2016-02-06

**Authors:** Shabnam Shamriz, Hamideh Ofoghi

**Affiliations:** Department of Biotechnology, Iranian Research Organization for Science and Technology, Tehran, Iran

**Keywords:** Malaria, Fusion protein, Molecular modeling, Molecular dynamics simulations

## Abstract

**Background:**

Malaria infection is still widespread in some parts of the world and threatens the lives of millions of people every year. Vaccines, especially oral vaccines are considered to be effective in reducing the burden of malaria morbidity and mortality. By using recombinant technology, suitable oral hosts could serve as antigen delivering vehicles in developing oral vaccines. This study was aimed towards designing and computational analysis of a fusion protein consisting of *Plasmodium falciparum* cell-traversal protein for ookinetes and sporozoites (*Pf*CelTOS) fused to human interleukin-2 (IL-2) and M cell-specific peptide ligand (Co1), as a step toward developing a vaccine candidate.

**Results:**

To our best knowledge, the three dimensional (3D) structure of CelTOS is not reported in protein database. Therefore, we carried out computational modeling and simulation in the hope of understanding the properties and structure of *Pf*CelTOS. Then we fused IL-2 to *Pf*CelTOS by a flexible linker and did in silico analysis to confirm the proper folding of each domain in the designed fusion protein. In the last step, Co1 ligand was added to the confirmed fusion structure using a rigid linker and computational analysis was performed to evaluate the final fusion construct. One structure out of five predicted by I-TASSER for *Pf*CelTOS and fusion constructs was selected based on the highest value for C-score. Molecular dynamics (MD) simulation analysis indicated that predicted structures are stable during the simulation. Ramchandran Plot analysis of *Pf*CelTOS and fusion constructs before and after MD simulation also represented that most residues were fallen in favorable regions.

**Conclusion:**

In silico study showed that Co1-(AEEEK)_3_- IL-2-(GGGGS)_3_-PfCelTOS construct has a constant structure and the selected linkers are effectively able to separate the domains. Therefore, data reported in this paper represents the first step toward developing of an oral vaccine candidate against malaria infection.

## Background

Infectious diseases are the leading cause of death all over the world [1, 2]. Malaria, also called plasmodium infection, is one of the most important human infectious diseases threatening the lives of millions of people every year. According to WHO malaria report, globally, about 3.2 billion people in 97 countries and territories are at risk of being infected with malaria, and 1.2 billion are at high risk [3]. Although the disease has been eradicated in most areas, it’s still widespread in some regions [3]. The biggest challenges in controlling malaria disease are the emergence of anti-malarial drug resistance and insecticide resistance in parasite and mosquito, respectively [4–6]. Therefore, there is an urgent need to develop novel intervention strategies such as vaccines to reduce the burden of malaria morbidity and mortality. Malaria is most common in poor and developing regions of the world and has a strong negative effect on economic growth [7]. Oral immunogenicity has opened new avenues for the development of vaccines potentially effective in reducing the burden of diseases especially in low-income and developing countries. Oral vaccines are Low cost, easily administered (needle-free), in most cases capable of being stored and transported without refrigeration (Non-Refrigerated) and painless [8]. In contrast to injected vaccines, oral vaccines target mucosal surfaces, which cause stimulation of systemic as well as mucosal immune responses [9–11]. Considering the fact that most pathogens enter the body through mucosal surfaces, mucosal immune system provides the first line of defense against invading bacteria and viruses. Therefore, the simultaneous induction of systemic and mucosal immunity offers an ideal strategy to fight infectious diseases. Despite many advantages that oral vaccines have, only limited numbers of them have been approved for human commercial use and yet significant challenges must be overcome to make oral vaccines closer to reality [12]. One of the challenges is the complexity of mucosal immune system that must discriminate between harmless and dangerous antigens. One way to overcome this problem is to use adjuvants. Oral adjuvants offer exciting possibilities for the formulation of oral vaccines [13–15].

During the past decades considerable progress in recombinant DNA technology has led to the development of fusion proteins. Fusion proteins are novel biomolecules obtained by genetically fusing two or more genes that originally code for separate proteins. Thereby fusion proteins have distinct functions derived from each of their domains. Fusion proteins have wide applications in industry and pharmaceutical protein production.

The general objective of the current study was the design and computational analysis of a fusion protein consisting of *Plasmodium falciparum* cell-traversal protein for ookinetes and sporozoites (*Pf*CelTOS) fused to human interleukin-2 (IL-2) and M cell-specific peptide ligand (Co1), as a step toward developing a candidate recombinant oral vaccine against malaria. CelTOS is a protein that mediates malarial invasion into its hosts (both vertebrate and mosquito) and is required for effective malaria infection [16–18]. On the other hand, CelTOS amino acid sequence is highly conserved among the *Plasmodium* species. CelTOS highly conserved amino acid sequence and vital role for malaria infection indicate its important function across all species [19]. Therefore, targeting the immune response to this highly conserved and crucial protein and interfering with its biological function could possibly result in protection against infection by heterologous species of *Plasmodium* [19].

In the designed construct, human interleukin 2 (IL-2) is thought to act as mucosal adjuvant. It should be considered that the type of desired immune response (cellular or humoral) will influence the choice of adjuvants for immunization regimens [20]. In case of malaria disease, parasites and infected red blood cells which activate dendritic cells through pattern recognition receptors (PRRs) are phagocytosed and their antigens are presented to T cells. PRR signaling causes the secretion of cytokines that initiate the inflammation that underlies malaria pathogenesis and direct TH1 cell to differentiate. TH1 cells help B cells differentiation and antibody secretion and also secrete IFN-γ, which activates macrophages that phagocytose opsonized parasites and infected red blood cells and subsequently kill them by NO- and O2-dependent pathways. Inflammation induces expression of endothelial adhesion molecules to which infected red blood cells bind. Finally the secretion of anti-inflammatory cytokines from macrophages and regulatory populations of T cells curtailed the Inflammation [21]. To the best of our understanding, adjuvants that increase the TH1 response are more appropriate in malaria vaccine development. Here we focus on human interleukin 2 (IL-2) as mucosal adjuvant. IL-2 has a central role in the cascade of events involved in immune responses and can function as a vaccine adjuvant to increase the immune response to vaccine immunogens [20, 22–26].

Specific advantages of IL-2 as adjuvant for malaria vaccine are as follow:

1. IL-2 stimulates T cells to secrete INF-γ. INF-γ is known as immune interferon due to activating macrophages and enhancing phagocytic activity which leads to activation of immune responses. On the other hand, INF-γ results in the expression of MHCI and MHCII molecules on the surface of infected cells and antigen presenting cells, respectively, therefore enhancing antigen presenting and immune system activity. In addition, INF-γ activates the secretion of IgG and its subclasses by effecting on B lymphocytes (The positive effect of INF-γ on immune responses to malaria parasite is discussed above).
2. Activation of T cells under the influence of IL-2 leads to the secretion of cytokines such as IL-10. Unlike many other interleukins, IL-10 inhibits macrophage activity and secretion of IL-1, IL-12 and TNF. IL-12 acts as inducer for INF-γ and is an effective factor in cellular and innate immune responses against intracellular microbes. Inhibition of IL-12 leads to inhibition of INF-γ production. In fact, IL-10 is well known as the inhibitor of immune responses, especially responses that are set up by macrophages, thereby immune responses are terminated. As mentioned above, the enhancement of inflammation and thereby endothelial adhesion of blood vessels is one of the outcomes of immune system responses to malaria parasite and malaria vaccine. In this case, repressive role of IL-10 prevents excessive inflammation.

Although reported studies have indicated the potential of cytokines as mucosal adjuvants, in order to increase the probability of vaccine candidate binding to intestinal cells, we adopted another strategy. Co1 is a peptide ligand that promotes the binding of ligand-fused antigen to human M-like cells and has also comparable levels of adjuvant activity to Cholera Toxin (CT) [27]. Conjugation of Co1 to the designed construct should result in delivery of antigen to M cells.

Since most of the biological functions of proteins depend upon their 3D structure, in designing multi domain recombinant proteins, proper folding, stability and interaction between domains must be considered. Fusion proteins are much more susceptible to misfolding than single-domain proteins due to the interaction between their different peptide domains. Therefore, in silico analysis of multi domain proteins is an indispensible stage in recombinant protein production projects.

Attempts to model the structure of proteins on the computer began about 30 years ago. Since that time our understanding of protein structure and dynamics has significantly increased and now Protein Data Bank (PDB) contains more than 10,000 high resolution protein structures. Valuable 3D models of a protein that has a clear sequence homology to known proteins can be predicted by homology modeling method. However, even in cases where there is no sequence homology, threading methods relate protein sequences to a library of known structures and predict the likely protein structure. The crystallographic structure of CelTOS has not been reported yet. In the present study, we carried out a molecular modeling study of *Pf*CelTOS protein and designed fusion proteins using iterative threading assembly refinement (I-TASSER) to obtain their 3D structures. Then energy minimization and molecular dynamics (MD) simulation were run to refine the models. The simulations of PfCelTOS and fusion proteins were performed for long time duration of 10 ns and the obtained structures were analyzed to verify further.

## Methods

### Sequence retrieval and fusion protein construction

The amino acid sequences of *Pf*CelTOS, human IL-2 and M cell-targeting ligand, Co1, were retrieved from UniProt (*Pf*CelTOS id: Q53UB8; human IL-2 id: P60568). Amino acid sequences were used to design fusion protein construct. The designed construct consisted of *Pf*CelTOS and human IL-2 mature parts linked together by a flexible linker, whereas Co1 is linked to this construct through a rigid (helical) linker.

### Primary structural analysis

Expasy’s Prot Param server [28] was used to study the physiochemical characters of *Pf*CelTOS and designed fusion constructs such as theoretical isoelectric point (pI), molecular weight, molecular formula, total number of positive and negative residues, instability index [29], aliphatic index [30] and grand average hydropathicity (GRAVY) [31]. The instability index provides an estimate of a protein’s stability in vitro. Proteins with instability index smaller than 40 are predicted as stable. A value above 40 indicates that the protein may be unstable.

The aliphatic index of a protein is regarded as a positive factor for the increase of thermostability of globular proteins and is mainly defined as the relative volume occupied by aliphatic side chains (alanine, valine, isoleucine and leucine). The GRAVY score is calculated as the sum of hydropathy values of all the amino acids, divided by the number of residues in the sequence.

### Secondary structure prediction

Secondary structure elements of *Pf*CelTOS and designed fusion proteins were determined using Phyre2 [32] and I-TASSER [33] online servers.

### Three-Dimensional (3D) model prediction

The 3D structure of *Pf*CelTOS and fusion proteins were modeled using I-TASSER online server. The raw amino acid sequences of *Pf*CelTOS and fusion proteins were uploaded in FASTA format to I-TASSER server. Tertiary structures were predicted in PDB format. The results generated five top models for each entry which the one with the highest confidence score (c-score) represented the best model and was the structure selected for this study [33].

### Structure Validation

Visualization was carried out by PyMOL version v1.7.2 software [34]. Traceable structural errors were proofed and global charges replaced by Vega ZZ version 3.0.5.12 software [35]. Ramachandran plots were calculated by RAMPAGE Ramachandran Plot Assessment [36]. Hydropathicity index were calculated by Marvin view version 14.7.7 software (http://www.chemaxon.com). Molecular dynamics were performed by GROMACS version 5.0.1 software [37, 38]. PDB file format were analyzed for MD approaches-molecular dynamics algorithm including an initial cubic salvation with 3 point simple water model, followed by ionization and neutralization of simulation cube with Na and Cl ions. Geometry optimization was performed with constrain method. This procedure continued with two separate temperature and pressure unconstrained global dynamics. Final unconstrained dynamics performed with coupled temperature (300° K) and pressure (1 bar) for 10 ns. A schematic sketch of molecular dynamics procedure is presented in Table 1.

**Table 1:** A schematic sketch of molecular dynamics procedure

Step	Constrain/Algorithm	Temperature	Pressure	Time	Neighborsearching	Electrostatic interactions (long-range)	Temperature coupling	Pressure coupling
Geometry Optimization	No	-	-	2 ns	grid	Particle Mesh Ewald (PME)	-	-
Temperature constant	No	300 K	-	0.1 ns	grid	PME	V-rescale	-
Pressure constant	No	-	1 bar	0.1 ns	grid	PME	-	Parrinello-Rahman
Total molecular dynamics	Yes/steep = steepest descent minimization	300 K	1 bar	10 ns	grid	PME	V-rescale	Parrinello-Rahman

### Analysis of simulation

**Fig. 1:**
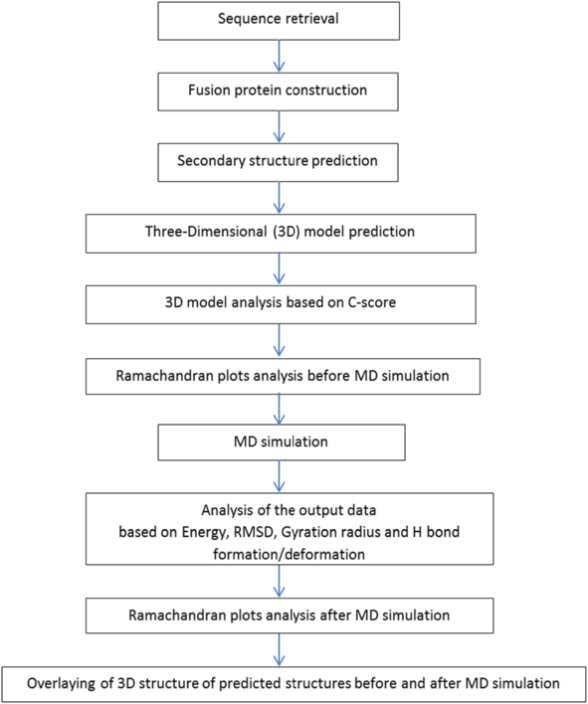
Screening procedure of modeled structures

After the simulation had finished, the output data were analyzed according to Energy (kinetic, potential & total energy), root-mean-square deviation (RMSD), Gyration radius and H bond formation/deformation. The following plot is represented as screening procedure of modeled structures (Fig. 1).

## Results and discussion

To our knowledge, the crystallographic structure of CelTOS has not been reported in the protein database. Therefore, *in silico* analysis of CelTOS 3D structure could be of benefit in predicting its probable structure. Computer prediction and simulation methods can be used to generate representative conformations of a molecule in equilibrium and provide a picture of the way in which a molecule changes from one configuration to another [39]. Therefore, we carried out computational modeling and simulation in the hope of understanding the properties and structure of *Pf*CelTOS. Then we fused IL-2 to *Pf*CelTOS by a flexible linker and did in silico analysis to confirm the proper folding of each domain in the designed fusion protein. In the last step, Co1 ligand was added to the confirmed fusion structure using a rigid linker and computational analysis was performed to evaluate the final fusion construct.

### Primary structure analysis

**Fig. 2:**
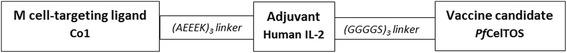
Schematic view of the designed fusion protein construct

The primary structure of *Pf*CelTOS (accession number Q53UB8) is composed of 182 amino acid residues, encoded by 546 nucleotides. A fragment of 9 amino acids at the N-terminal is a signal peptide and the next 173 amino acids fragment is a mature peptide. Human IL-2 (accession number P60568) composed of 153 amino acid residues, encoded by 459 nucleotides. A fragment of 20 amino acids at the N-terminal was found to be a signal peptide and the remaining 133 amino acids fragment was a mature peptide [40]. The 173 amino acids fragment of *Pf*CelTOS (mature part) and 133 amino acids fragment of IL-2 were used to design the fusion protein construct. The length of the designed construct is 321 amino acids, which within 1 to 133 is IL-2. Amino acids 133 to 148 (15 aa) are linker sequence, which is (GGGGS)_3_. Amino acids 149 to 321 (173 aa) are *Pf*CelTOS. Based on previous studies, N-terminal of IL-2 and C-terminal of *Pf*CelTOS seems more important in their biological function than the other end. Therefore we designed the fusion construct in a way to make free the N- and C-terminals of IL-2 and *Pf*CelTOS, respectively. The Co1 ligand peptide was added to the computationally confirmed structure of IL-2-(GGGGS)_3_-PfCelTOS. To present the Co1 ligand to human M-like cells better, a rigid linker ((AEEEK)_3_) was used between Co1 ligand and designed fusion protein and the new construct was designed as follow: Co1-(AEEEK)_3_- IL-2-(GGGGS)_3_-*Pf*CelTOS. Figure 2 shows schematic view of designed fusion protein construct.

The primary structural features of *Pf*CelTOS and two designed fusion constructs determined by Prot Param are summarized in Table 2. Prot Param is a tool which allows the computation of various physical and chemical parameters for a given protein. The calculated isoelectric points (pI) for PfCelTOS, IL-2-(GGGGS)_3_-PfCelTOS and Co1-(AEEEK)_3_- IL-2-(GGGGS)_3_-PfCelTOS were 4.58, 5.11 and 5.32, respectively, suggesting the presence of more negatively charged residues. Although, proteins were found to be stable due to their high aliphatic index, an instability index of more than 40 suggests instability and thus predicted that the proteins were thermally unstable. The value of aliphatic index for *Pf*CelTOS, IL-2-(GGGGS)_3_-*Pf*CelTOS and Co1-(AEEEK)_3_- IL-2-(GGGGS)_3_-*Pf*CelTOS were 89.48, 91.06 and 89.11, respectively, while that of instability index were 41.67, 48.01 and 49.75. GRAVY (Grand average hydropathy) values of *Pf*CelTOS, IL-2-(GGGGS)_3_-*Pf*CelTOS and Co1-(AEEEK)_3_- IL-2-(GGGGS)_3_-*Pf*CelTOS were −0.106, −0.151 and −0.172, respectively, suggesting a hydrophilicity pattern with better interaction with water.

**Table 2:** Properties of *Pf*CelTOS, human IL-2 and designed constructs determined by ProtParam

Protein	*Pf*CelTOS	Fusion protein1	Fusion protein2
		(IL-2-linker-*Pf*CelTOS)	(Co1-linker-IL-2-linker-*Pf*CelTOS)
No. of amino acids	173	321	348
Molecular weight	19087.4	35433.2	38176.4
Theoretical pI	4.58	5.11	5.32
Total No. of negatively charged residues (Asp + Glu)	23	38	41
Total No. of positively charged residues (Arg + Lys)	13	28	32
Formula	C_855_H_1326_N_212_O_274_S_4_	C_1581_H_2495_N_405_O_494_S_11_	C_1703_H_2691_N_441_O_530_S_11_
Instability index	41.67	48.01	49.75
Aliphatic index	89.48	91.06	89.11
Grand average of hydropathicity (GRAVY)	−0.106	−0.151	−0.172

### Secondary structure prediction

Among protein structure prediction software, the on-line servers seem more appropriate since they have access to all structures available in databases. Secondary structure of *Pf*CelTOS and designed fusion constructs were predicted by Phyre2 and I-TASSER servers. phyre2 (Protein Homology/AnalogY Recognition Engine; pronounced as ‘fire’) is among the most popular methods for protein structure prediction having been cited over 1500 times and has been designed to ensure a user-friendly interface for users inexpert in protein structure prediction methods. Both programs established that *Pf*CelTOS structure is predominantly made of α-helices and coil–coils and there was no significant strand in *Pf*CelTOS. The location of rigid linker fragment within amino acids 13–27 in Co1-(AEEEK)_3_- IL-2-(GGGGS)_3_-*Pf*CelTOS was shown by helical structures. Phyre2 prediction and analysis of secondary structure of proteins is shown in Table 3. In case of the fusion constructs also, amount of α-helices exceeded β-sheets. The presence of only 5 % β-sheets is related IL-2 structure (a bend created by proline residue at position 47 0f IL-2).

**Table 3:** Phyre2 prediction and analysis of secondary structure

Protein	α-helix	β sheets	Coils
*Pf*CelTOS	79 %	0 %	25 %
IL-2-linker-*Pf*CelTOS	65 %	5 %	24 %
Co1-linker-IL-2-linker-*Pf*CelTOS	63 %	5 %	27 %

### Three-Dimensional (3D) structure prediction

Prediction of 3D structure was performed by I-TASSER online server using the best aligned template obtained by searching against Protein Data Bank database. I-TASSER (Iterative Threading ASSEmbly Refinement) server (as ‘Zhang-Server’) is a web-based service for the prediction of protein structures and functions. It’s free for academic users and allows them to automatically generate high-quality models of 3D structure of proteins from their amino acid sequences. It detects structure templates from PDB by threading technique. I-TASSER is one of the most successful servers in the community-wide CASP (Critical Assessment of protein Structure Prediction) experiments and was ranked as the No 1 server for protein structure prediction in recent CASP7 CASP8, CASP9, CASP10, and CASP11 experiments.

**Fig. 3:**
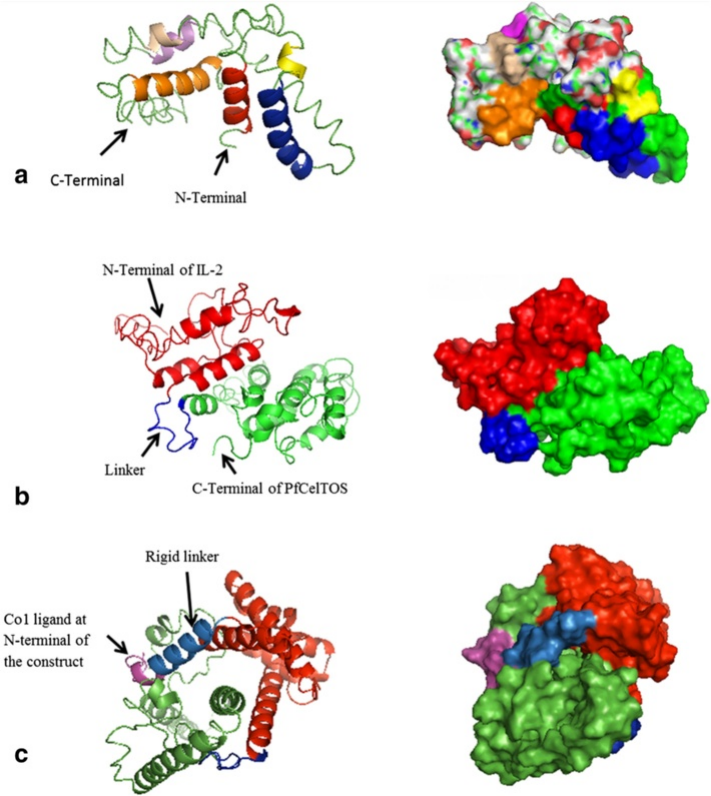
I-TASSER predicted 3D structures of (**a**) *Pf*CelTOS, (**b**) IL-2-(GGGGS)_3_-*Pf*CelTOS and (**c**)Co1-(AEEEK)_3_- IL-2-(GGGGS)_3_-*Pf*CelTOS

3D structure prediction by I-TASSER generates five top models with C-scores ranging from −5 to 2. The one with the highest C-score represents the best model. The highest value for predicted models of *Pf*CelTOS, IL-2-(GGGGS)_3_-*Pf*CelTOS and Co1-(AEEEK)_3_- IL-2-(GGGGS)_3_-*Pf*CelTOS were −2.94, −2.23 and −2.67, respectively. These models were selected for this study. The best selected model for *Pf*CelTOS based on C-score represented that the 3D structure is consistent with secondary structure elements generated by Phyre2. Based on Phyre2 SS confidence, amino acids 3–15, 25–28, 32–47, 85–112 and 122–145 are α-helices with high confidence, which are also predicted as α-helix structures in 3D model of *Pf*CelTOS generated by I-TASSER (shown in different colors in Fig. 3). It is important to note that further fusion protein constructs linked by other flexible linkers were designed but the one with (GGGGS)_3_ linker resulted in the best 3D structure based on C-score. Predicted model for IL-2-(GGGGS)_3_-*Pf*CelTOS showed a protein with two separate domains linked together through a small linker. Glycine-rich peptides confer flexibility, which allows the domains to move independently of one another while maintaining their individual three dimensional shapes [41]. 3D structure prediction of Co1-(AEEEK)_3_- IL-2-(GGGGS)_3_-*Pf*CelTOS showed a protein with three domains linked together by two small linkers. (AEEEK)_3_ rigid linker keeps domains apart and ensure a relatively rigid separation of the domain and carrier [41]. The pre-simulated 3D structures of PfCelTOS and fusion proteins are shown in Fig. 3.

### Structural model refinement

The structural refinement was carried out using molecular dynamics simulation as described in methods. Simulation acts as a bridge between theory and experiment. Indeed we test a theory by conducting a simulation using the same or computationally predicted models and provide a guess at the possible interactions between molecules [39, 42]. Gromacs is a molecular dynamics application developed by Groningen University. Gromacs is able to work in the operating system Linux. The main ability of Gromacs is to perform MD simulation and minimization energy. However, Gromacs does not work alone and should interact with PyMOL and Grace. PyMOL is an application to visualize molecule structure and Grace is an application in Linux to display graphs. Both applications support analysis of MD simulation.

**Fig. 4:**
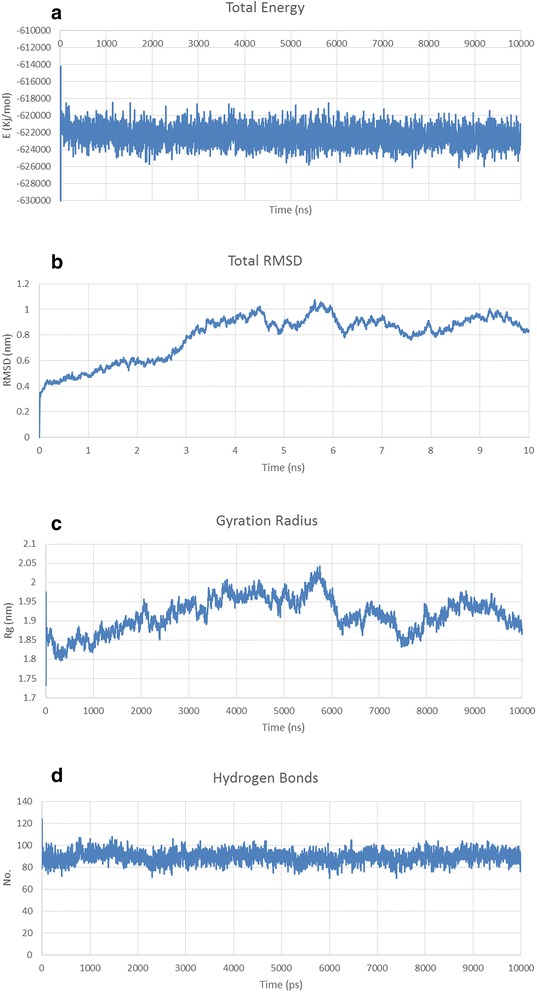
Structural model refinement analysis of *Pf*CelTOS predicted structure. **a** Energy plots for the molecular dynamics simulation of *Pf*CelTOS predicted structure. **b** Total RMSD, **c** the radius of gyration and **d** Hydrogen bond formation/deformation of *Pf*CelTOS predicted structure. RMSD of *Pf*CelTOS stable conformation observed after the extension of a 10 ns simulation run

**Fig. 5:**
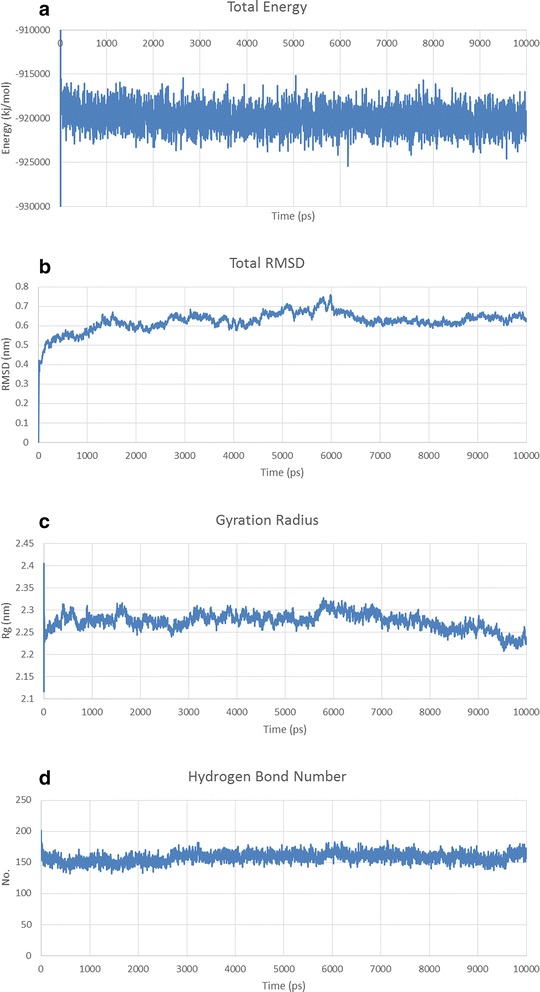
Structural model refinement analysis of IL-2-(GGGGS)_3_-*Pf*CelTOS predicted structure. **a** Total, Potential and Kinetic energies for MD simulation of IL-2-(GGGGS)_3_-*Pf*CelTOS predicted structure. **b** Total RMSD, **c** the radius of gyration and **d** Hydrogen bond formation/deformation of IL-2-(GGGGS)_3_-*Pf*CelTOS predicted structure

**Fig. 6:**
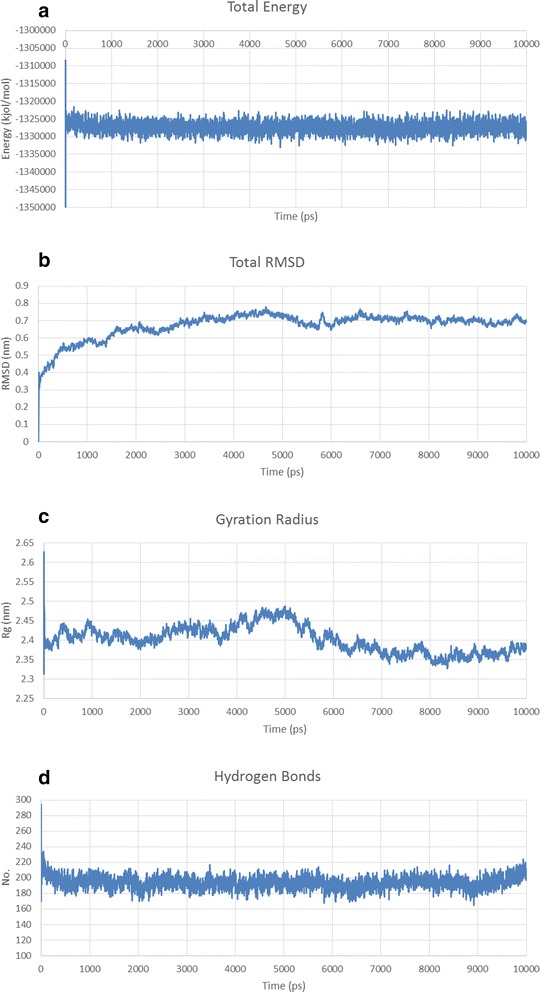
Structural model refinement analysis of Co1-(AEEEK)_3_- IL-2-(GGGGS)_3_-*Pf*CelTOS predicted structure. **a** Total, Potential and Kinetic energies for MD simulation of Co1-(AEEEK)_3_- IL-2-(GGGGS)_3_-*Pf*CelTOS Structural model refinement analysis. **b** Total RMSD, **c** the radius of gyration and **d** Hydrogen bond formation/deformation of Co1-(AEEEK)_3_- IL-2-(GGGGS)_3_-*Pf*CelTOS predicted structure

The MD simulation output data were analyzed based on Energy (kinetic, potential & total energy), RMSD [43], Gyration radius [44] and H bond formation/deformation [45]. The total energy should be constant throughout the simulation process, as it is the sum of kinetic and potential energy of the molecules. Kinetic energy should be constant or following a decreasing trend since the constant increasing of kinetic energy level reflects the general confusion of protein structure. Potential energy level should be increasing or constant to show the stability of structure. RMSD is the measure of the average distance between the backbone atoms of proteins. The structural refinement was carried out using molecular dynamics simulation over the equilibration course and exhibits RMSD plots for predicted models of *Pf*CelTOS and fusion constructs that flattens during 10 ns. RMSD plots indicated that the proteins are stable during the simulation. Gyration radius is an indicative of the level of compaction in the structure*,* i.e., how folded or unfolded is the protein. H bond formation/deformation factor represents the number of hydrogen bonds formed or broken during molecular simulation. During 10 ns of simulation, this number was almost constant indicating the stability of the molecular structures. Figures 4, 5 and 6 represents simulation analysis of *Pf*CelTOS, IL-2-(GGGGS)_3_-*Pf*CelTOS and Co1-(AEEEK)_3_- IL-2-(GGGGS)_3_-*Pf*CelTOS, respectively, that validated the accuracy of our designed fusion protein structure.

### Structure validation of predicted models

**Fig. 7:**
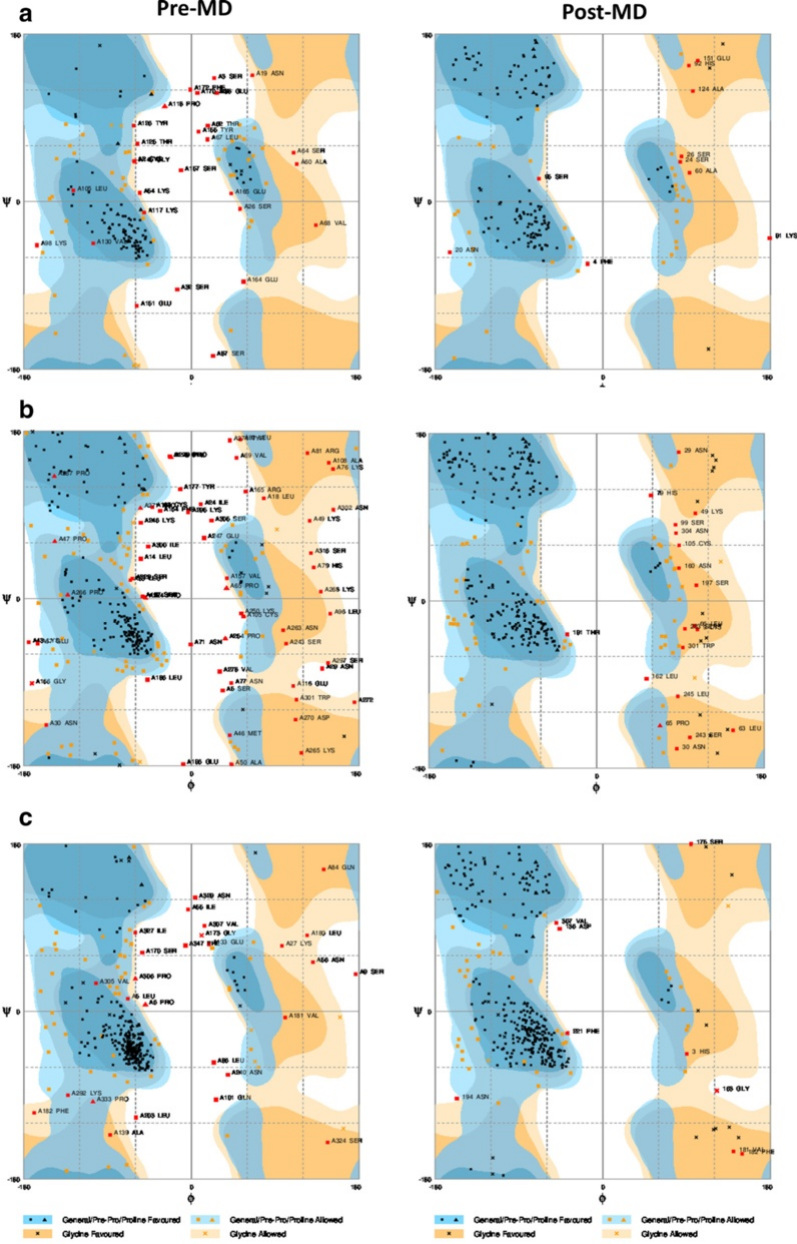
Comparative diagram depicting Ramchandran Plot analysis of *Pf*CelTOS an fusion proteins before and after molecular dynamic simulations (**a**) *Pf*CelTOS (**b**) IL-2-(GGGGS)_3_-*Pf*CelTOS (**c**) Co1-(AEEEK)_3_- IL-2-(GGGGS)_3_-*Pf*CelTOS. The plots were analyzed by RAMPAGE Ramachandran Plot Assessment. The general favored region and Pro-Pro favored region are indicated with dark blue color. The general allowed region and Pro-Pro allowed region is shown in pale blue. The Glycine favored and allowed regions are shown in dark and pale orange, respectively. The disallowed region is in white color

To validate the modeled structures, Ramachandran maps were drawn before as well as after MD simulation and structures were analyzed using RAMPAGE Ramachandran Plot Assessment. The Ramchandran plot analysis obtained by RAMPAGE Ramachandran Plot Assessment is summarized in Table 4 and the plots are provided in Fig. 7. This provides an insight into the correctness of the modeled structures. As it’s obvious Co1-(AEEEK)_3_- IL-2-(GGGGS)_3_-*Pf*CelTOS has a well modeled structure in terms of RAMPAGE Ramachandran Plot Assessment.

**Table 4:** The Ramchandran plot analysis of the proteins before and after MD simulation obtained by RAMPAGE Ramachandran Plot Assessment

Proteins		Favored region	Allowed region	Outlier region
*Pf*CelTOS	Pre-MD	57.3 %	26.3 %	16.4 %
	Post-MD	74.7 %	19.4 %	5.9 %
IL-2-(GGGGS)_3_-*Pf*CelTOS	Pre-MD	58.3 %	22.9 %	18.8 %
	Post-MD	76.4 %	17.6 %	6.0 %
Co1-(AEEEK)_3_-IL-2-(GGGGS)_3_-*Pf*CelTOS	Pre-MD	76.3 %	15.9 %	7.8 %
	Post-MD	85.8 %	11.6 %	2.6 %

### Comparison of predicted structures before and after MD simulation

**Fig. 8:**
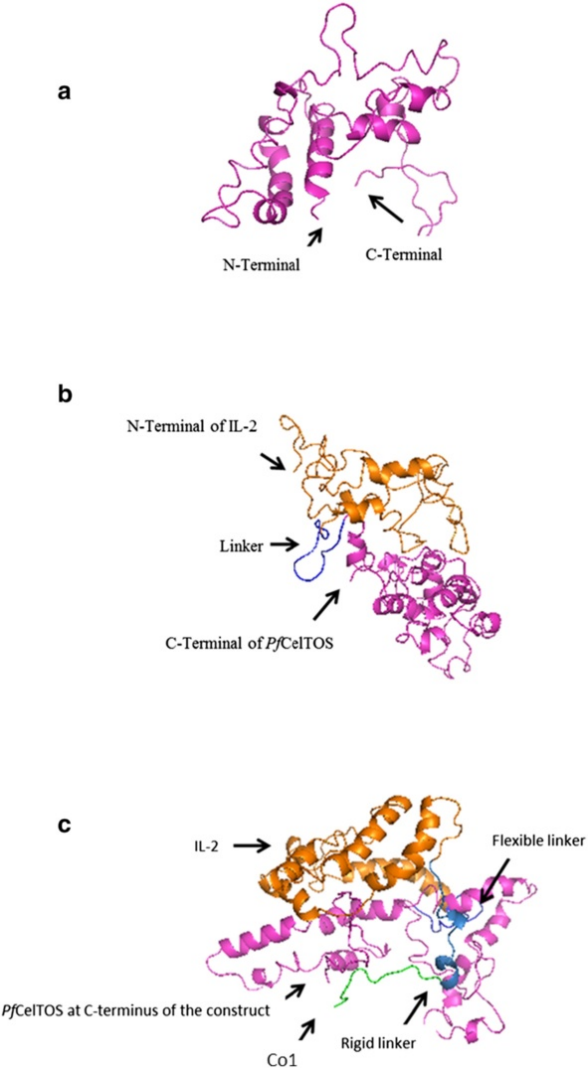
3D structures of (**a**) *Pf*CelTOS, (**b**) IL-2-(GGGGS)_3_-*Pf*CelTOS and (**c**) Co1-(AEEEK)_3_- IL-2-(GGGGS)_3_-*Pf*CelTOS after molecular dynamics simulations

**Fig. 9:**
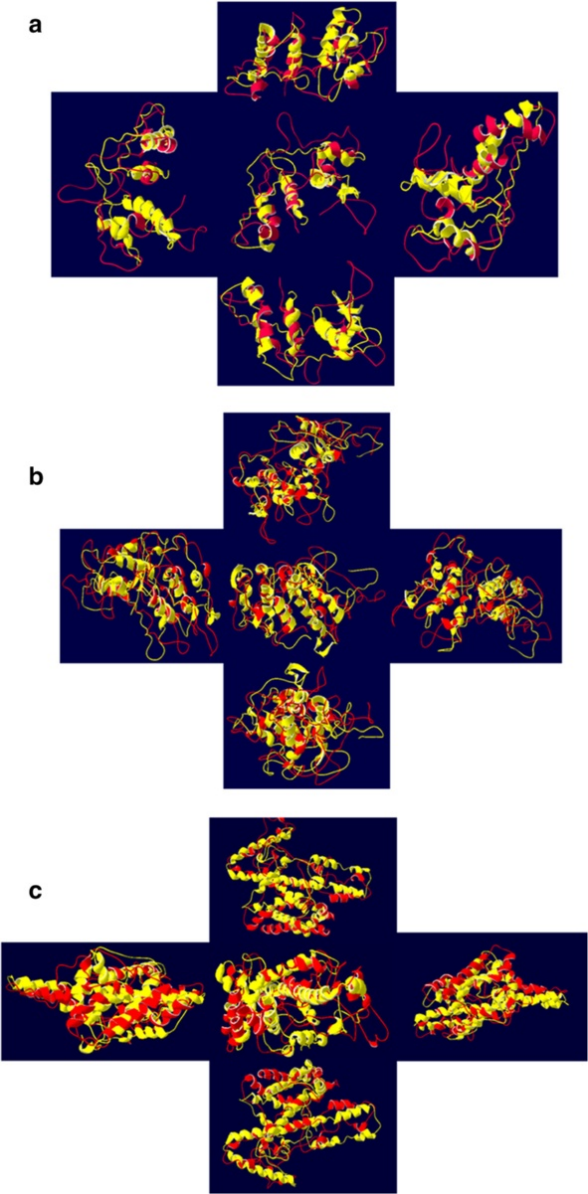
Structural comparison of predicted models for (**a**) *Pf*CelTOS (**b**) IL-2-(GGGGS)_3_-*Pf*CelTOS (**c**) Co1-(AEEEK)_3_- IL-2-(GGGGS)_3_-*Pf*CelTOS, obtained before (shown in *yellow*) and after molecular dynamics simulations (shown in *red*) from different views

**Fig. 10:**
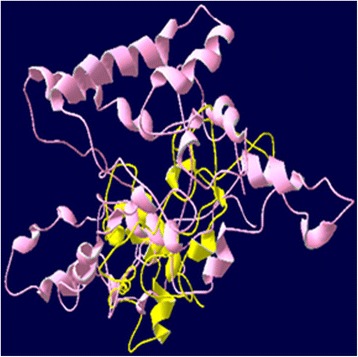
Structural comparison of predicted models of *Pf*CelTOS and Co1-(AEEEK)_3_- IL-2-(GGGGS)_3_-*Pf*CelTOS, obtained after molecular dynamics simulations

3D Structures of *Pf*CelTOS and fusion constructs were also investigated after MD simulation (Fig. 8). Overlaying of 3D structure of *Pf*CelTOS and designed fusion proteins before and after MD simulation showed that the predicted structures are constantly stable and the selected linkers are able to separate the domains of designed fusion constructs effectively. As it’s obvious, rigid linker ensures the separation of domains and carrier and leads to the better presentation of construct to human M-like cells (Fig. 9). Structural comparison of predicted models of *Pf*CelTOS and Co1-(AEEEK)_3−_ IL-2-(GGGGS)_*3*_-*Pf*CelTOS, obtained after molecular dynamics simulations is shown in Fig. 10. This comparison indicates the proper folding of *Pf*CelTOS in fusion construct.

## Conclusion

The procedure of our study (as shown in Fig. 1) helps to rapid analysis of designed fusion constructs before initializing the recombinant fusion protein lab experiments. As it is obvious, this procedure is fast, inexpensive (since all the servers are free) and simple, especially for inexpert users in this field.

In silico study of Co1-(AEEEK)_3_- IL-2-(GGGGS)_3_-*Pf*CelTOS structure through this procedure revealed that designed construction is suitable for fusion protein expression in edible host cells for oral delivery. Flexible linker separates *Pf*CelTOS and IL-2 domains effectively to maintain their proper individual three dimensional structures and allows them to move independently of one another. On the other hand, rigid linker ensures the separation of fusion protein and carrier and lead to the better presentation of fusion construct to human M-like cells. Therefore, data reported in this paper represents the first step toward developing of an oral vaccine candidate against malaria infection.

## References

[1] Debast S, Bauer M, Kuijper E, European Society of Clinical Microbiology and Infectious Diseases (2014). European society of clinical microbiology and infectious diseases: update of the treatment guidance document for clostridium difficile infection. Clin Microbiol Infect.

[2] Brower J, Chalk P. The global threat of new and reemerging infectious diseases: reconciling US national security and public health policy. Rand Corporation; 2003

[3] WHO. World malaria report 2013. World Health Organization; 2014

[4] O’Brien C, Henrich P, Passi N, Fridock D. Recent clinical and molecular insights into emerging artemisinin resistance in Plasmodium falciparum. Curr Opin Infect Dis. 2011;24(6):570. 10.1097/QCO.0b013e32834cd3ed PMC3268008 22001944

[5] McNamara C, Winzeler EA (2011). Target identification and validation of novel antimalarials. Future Microbiol.

[6] Ranson H, N’Guessan R, Lines J, Moiroux N, Nkuni Z, Corbel V. Pyrethroid resistance in African anopheline mosquitoes: what are the implications for malaria control? Trends Parasitol. 2011;27(2):91–8. 10.1016/j.pt.2010.08.004 20843745

[7] Ingstad B, Munthali A, Braathen S, Grut L. The evil circle of poverty: a qualitative study of malaria and disability. Malar J. 2012;11(11):15. 10.1186/1475-2875-11-15 PMC3295708 22236358

[8] Dhama K (2013). Plant based oral vaccines for human and animal pathogens-a new era of prophylaxis: Current and future prospective. J Exp Biol Agric Sci.

[9] Lycke N (2012). Recent progress in mucosal vaccine development: potential and limitations. Nat Rev Immunol.

[10] Czerkinsky C, Holmgren J. Mucosal delivery routes for optimal immunization: targeting immunity to the right tissues. *Mucosal* Vaccines, Springer. 2010;354:1-18. 10.1007/82_2010_112 21053117

[11] Pasetti MF (2011). Immunology of gut mucosal vaccines. Immunol Rev.

[12] Price JD, Tarbell KV (2015). The role of dendritic cell subsets and innate immunity in the pathogenesis of type 1 diabetes and other autoimmune diseases. Front Immunol.

[13] Kraehenbuhl J-P, Neutra MR (2013). Mucosal vaccines: where do we stand?. Curr Top Med Chem.

[14] Rhee JH, Lee SE, Kim SY (2012). Mucosal vaccine adjuvants update. Clin Exp Vaccine Res.

[15] Newsted D, Fallahi F, Golshani A, Azizi A. Advances and challenges in mucosal adjuvant technology. Vaccine. 2015;33(21):2399–405. 10.1016/j.vaccine.2015.03.096 25865473

[16] Kariu T, Ishino T, Yano K, Chinzei Y, Yuda M. CelTOS, a novel malarial protein that mediates transmission to mosquito and vertebrate hosts. Mol Microbiol. 2006;59(5):1369–79. 10.1111/j.1365-2958.2005.05024.x 16468982

[17] Bergmann-Leitner E, Legler P, Savranskaya T, Ockenhouse C, Angov E. Cellular and humoral immune effector mechanisms required for sterile protection against sporozoite challenge induced with the novel malaria vaccine candidate CelTOS. Vaccine. 2011;29(35):5940–9. 10.1016/j.vaccine.2011.06.053 21722682

[18] Bergmann-Leitner E, Li Q, Caridha D, O’Neil M, Ockenhouse C, Hickman M, Angov E. Protective immune mechanisms against pre-erythrocytic forms of Plasmodium berghei depend on the target antigen. Trials Vaccinology. 2014;3:6–10.

[19] Bergmann-Leitner E, Mease R, Vega P, Savranskaya T, Polhemus M, Ockenhouse C, Angov E. Immunization with pre-erythrocytic antigen CelTOS from Plasmodium falciparum elicits cross-species protection against heterologous challenge with Plasmodium berghei. Plos one. 2010;5(8):1-9. 10.1371/journal.pone.0012294 PMC2924390 20808868

[20] Giedlin M.A. Cytokines as vaccine adjuvants: the use of interleukin-2. Vaccine Adjuvants, Springer. 2000;42:283–297.

[21] Riley EM, Stewart VA (2013). Immune mechanisms in malaria: new insights in vaccine development. Nature Med.

[22] Nohria A, Rubin RH. Cytokines as potential vaccine adjuvants. chapter: Cytokines in the Treatment of Infectious Diseases, Springer. 1995;261–9.

[23] Derosa D, Sordillo L (1997). Efficacy of a Bovine Staphylococcus aureus Vaccine using Interleukin‐2 as an Adjuvant. J Veterinary Med Ser B.

[24] Zhang H, Qiu Y, Zhao Y, Liu X, Liu M, Yu A. Immunogenicity of oral vaccination with Lactococcus lactis derived vaccine candidate antigen (UreB) of Helicobacter pylori fused with the human interleukin 2 as adjuvant. Mol Cell Probes. 2014;28(1):25–30. 10.1016/j.mcp.2013.08.003 24036137

[25] Nunberg J, Doyle M, York S, York C. Interleukin 2 acts as an adjuvant to increase the potency of inactivated rabies virus vaccine. Proc Natl Acad Sci. 1989;86(11):4240–3. 10.1073/pnas.86.11.4240 PMC287426 2786210

[26] Overwijk WW, Theoret MR, Restifo NP. The future of interleukin-2: enhancing therapeutic anticancer vaccines. Cancer J Sci Am. 2000;6(Suppl 1):S76–S80. PMC2538796 10685664

[27] Kim S, Seo K, Kim J, Lee K, Jang Y. The M cell-targeting ligand promotes antigen delivery and induces antigen-specific immune responses in mucosal vaccination. J Immunol. 2010;185(10):5787–95. 10.4049/jimmunol.0903184 20952686

[28] Gasteiger E, Hoogland C, Gattiker A, Duvaud S, Wilkins M, Appel R, Bairoch A. Protein identification and analysis tools on the ExPASy server. Springer; 2005. 10.1385/1-59259-584-7:531 10027275

[29] Guruprasad K, Reddy BB, Pandit MW (1990). Correlation between stability of a protein and its dipeptide composition: a novel approach for predicting in vivo stability of a protein from its primary sequence. Protein Eng.

[30] Atsushi I (1980). Thermostability and aliphatic index of globular proteins. J Biochem.

[31] Kyte J, Doolittle RF (1982). A simple method for displaying the hydropathic character of a protein. J Mol Biol.

[32] Kelley L, Mezulis S, Yates C, Wass M, Sternberg M. The Phyre2 web portal for protein modeling, prediction and analysis. Nat Protoc. 2015;10(6):845–58. 10.1038/nprot.2015.053 PMC5298202 25950237

[33] Yang J, Yan R, Roy A, Xu D, Poisson J, Zhang Y. The I-TASSER Suite: protein structure and function prediction. Nat Methods. 2015;12(1):7–8. 10.1038/nmeth.3213 PMC4428668 25549265

[34] DeLano WL (2002). The PyMOL molecular graphics system.

[35] Pedretti A, Villa L, Vistoli G (2004). VEGA-an open platform to develop chemo-bio-informatics applications, using plug-in architecture and script programming. J Comput Aided Mol Des.

[36] Lovell S, Davis I, Arendall III B, Bakker P, Word M, Prisant M, Richardson J, Richardson D. Structure validation by Cα geometry: ϕ, ψ and Cβ deviation. Proteins: Struct, Funct, Bioinf. 2003;50(3):437–50. 10.1002/prot.10286 12557186

[37] Hess BD, van der Spoel, Lindahl E. GROMACS Groningen Machine for Chemical Simulations*.* User Manual. Version 4.5. 4.

[38] Abraham M, Hess B, Spoel D, Lindahl E. GROMACS User Manual version 5.0.1. 2014. http://www.gromacs.org.

[39] Allen MP (2004). Introduction to molecular dynamics simulation. Computational Soft Matter: Synthetic Polymers Proteins.

[40] Ju G, Collins L, Kaffka KL, Tsien WH, Chizzonite R, Crow R, Bhatt R, Kilian PL. Structure-function analysis of human interleukin-2. Identification of amino acid residues required for biological activity. J Biol Chem. 1987;262(12):5723–31. 3106342

[41] Wriggers W, Chakravarty S, Jennings PA (2005). Control of protein functional dynamics by peptide linkers. Pept Sci.

[42] Astuti A, Mutiara A. Performance Analysis on Molecular Dynamics Simulation of Protein Using GROMACS*.* arXiv preprint arXiv:0912.0893. 2009.

[43] Maiorov VN, Crippen GM (1994). Significance of root-mean-square deviation in comparing three-dimensional structures of globular proteins. J Mol Biol.

[44] Lobanov MY, Bogatyreva N, Galzitskaya O (2008). Radius of gyration as an indicator of protein structure compactness. Mol Biol.

[45] Konermann L, Wilson DJ, Simmons DA. Stopped-flow Electrospray Ionization Mass Spectrometry. Methods in Protein Structure and Stability Analysis: NMR and EPR Spectroscopies, Mass-Spectrometry and Protein Imaging. Chapter 2.1. pp. 95-119

